# Environmental Transmission of the Gut Symbiont *Burkholderia* to Phloem-Feeding *Blissus insularis*

**DOI:** 10.1371/journal.pone.0161699

**Published:** 2016-08-22

**Authors:** Yao Xu, Eileen A. Buss, Drion G. Boucias

**Affiliations:** Department of Entomology and Nematology, University of Florida, Gainesville, Florida, United States of America; Pusan National University, REPUBLIC OF KOREA

## Abstract

The plant-phloem-feeding *Blissus insularis* possesses specialized midgut crypts, which harbor a dense population of the exocellular bacterial symbiont *Burkholderia*. Most individual *B*. *insularis* harbor a single *Burkholderia* ribotype in their midgut crypts; however, a diverse *Burkholderia* community exists within a host population. To understand the mechanism underlying the consistent occurrence of various *Burkholderia* in *B*. *insularis* and their specific association, we investigated potential gut symbiont transmission routes. PCR amplification detected a low titer of *Burkholderia* in adult reproductive tracts; however, fluorescence *in situ* hybridization assays failed to produce detectable signals in these tracts. Furthermore, no *Burkholderia*-specific PCR signals were detected in eggs and neonates, suggesting that it is unlikely that *B*. *insularis* prenatally transmits gut symbionts via ovarioles. In rearing experiments, most nymphs reared on St. Augustinegrass treated with cultured *Burkholderia* harbored the cultured *Burkholderia* strains. *Burkholderia* was detected in the untreated host grass of *B*. *insularis*, and most nymphs reared on untreated grass harbored a *Burkholderia* ribotype that was closely related to a plant-associated *Burkholderia* strain. These findings revealed that *B*. *insularis* neonates acquired *Burkholderia* primarily from the environment (*i*.*e*., plants and soils), even though the possibility of acquisition via egg surface cannot be excluded. In addition, our study explains how the diverse *Burkholderia* symbiont community in *B*. *insularis* populations can be maintained.

## Introduction

In heteropterans, exocellular gut symbiont bacteria can be acquired by aposymbiotic neonates from symbiont-contaminated egg chorion [1–4], specialized egg capsules [5,6], and symbiont-containing feces [7–9]. These transmission routes rely primarily on the symbiont-infected mother, whose gut symbionts are transmitted postnatally to their offspring. In many cases, these vertically transmitted exocellular gut symbionts belong to Gammaproteobacteria [3–5,9], a sister group to the obligate endocellular symbionts that includes members such as *Buchnera* in aphids, *Carsonella* in psyllids, *Baumannia* in sharpshooters, and *Wigglesworthia* in tsetse flies [10]. In addition to vertical transmission, transmission via the ambient environment is also found in a heteropteran species, *Riptortus pedestris* (F.), whose aposymbiotic neonates acquire orally the free-living bacteria *Burkholderia* from plants and soils in every generation without a vertical transmission mechanism [11]. Moreover, the coexistence of environmental and vertical transmissions can occur, as reported in a blissid species, *Cavelerius saccharivorus* (Okajima), and that may represent an intermediate stage in the evolutionary process of symbiont transmission mechanisms from environmental acquisition to strictly vertical transmission [12].

The Southern chinch bug, *Blissus insularis* Barber (Hemiptera: Lygaeoidea: Blissidae), is a plant-phloem-feeding pest of St. Augustinegrass, *Stenotaphrum secundatum* (Walter) Kuntze [13,14]. This chinch bug harbors a high density of exocellular *Burkholderia* within its tubular midgut crypts [15,16]. Phylogenetic analyses of 16S rRNA gene sequences derived from both field-collected [15] and laboratory-reared [16] *B*. *insularis* midgut crypts revealed that these symbiotic *Burkholderia* ribotypes did not reflect host populations and were randomly distributed into the stink bug-associated beneficial and environmental (SBE), the plant-associated beneficial and environmental (PBE), and the *Burkholderia cepacia* complex (BCC) clades, suggesting an environmental transmission mechanism of gut symbionts in *B*. *insularis*. Furthermore, *Burkholderia* was detected by quantitative PCR (qPCR) amplifications at a low titer (2 × 10^4^ *Burkholderia* 16S rRNA gene copies per egg) in *B*. *insularis* eggs [15], which implied a co-existence of environmental and vertical transmissions of *Burkholderia* in *B*. *insularis*. However, no experiments were conducted to validate this hypothesis. Hence, in this study, the female reproductive tracts of *B*. *insularis* individuals from two laboratory colonies were examined to detect *Burkholderia* and to determine their bacterial composition using 16S rRNA gene sequencing. Then, the proposed vertical transmission via egg surface contamination [15] was examined using *B*. *insularis* eggs and newly hatched neonates, with or without egg surface sterilization. Additional rearing experiments were conducted to explore potential environmental transmission by inoculating the cultured *Burkholderia* [16] on the egg chorion and/or on the host plants and determining the presence of inoculated *Burkholderia* in *B*. *insularis* midgut crypts.

## Materials and Methods

### Insect dissection and DNA extraction

Twenty-four adult *B*. *insularis* females from two laboratory colonies (13 from BiR colony, Bi01_R to Bi13_R; 11 from BiS colony, Bi01_S to Bi11_S), maintained over multiple generations using cut St. Augustinegrass and fresh, surface-sterilized yellow corn cobs as a food source at 27 ± 1°C with a 14:10 (L:D) h photoperiod [16,17], were surface-sterilized. Their dissected midgut crypts (denoted as Bi01MC_R to Bi13MC_R and Bi01MC_S to Bi11MC_S) and reproductive tracts (denoted as Bi01RT_R to Bi13RT_R and Bi01RT_S to Bi11RT_S) were subjected to genomic DNA extraction using the MasterPure™ Yeast DNA Purification Kit (Epicentre, Madison, WI), as described previously [16]. To minimize potential microbial cross-contamination, reproductive tracts were removed before dissection of crypts.

### Sequencing and analyses of 16S rRNA genes

The 16S rRNA gene sequences of bacteria associated with 24 crypt and 24 reproductive-tract DNA preparations were generated using universal (10F and 1507R) primers [18]. The 1.5-kb PCR amplicons were purified and subjected to bi-directional Sanger sequencing [16]. Sequencing revealed a mix of 16S rRNA gene reads in the reproductive tracts (see Results section below: Bacterial ribotypes in midgut crypts and reproductive tracts of *B. insularis*). To resolve the bacterial composition in the reproductive tracts, two pools of the purified universal 16S rRNA gene amplicons prepared from four BiR and four BiS reproductive-tract preparations were cloned separately into the pCR8/GW/TOPO^®^ vector (Invitrogen, Life Technologies, Grand Island, NY) and amplified in One Shot^®^ Mach1-T1 Chemically Competent *E*. *coli* cells (Invitrogen). Colony PCR screenings using the vector-specific M13 forward and reverse primers were conducted to select positive clones. For each cloning event, 48 positive BiR and 48 positive BiS clones were subjected to rolling-circle amplification and Sanger sequencing (ICBR Sequencing Core, University of Florida) using the plasmid primer GW1 (Invitrogen).

To detect the *Burkholderia* 16S rRNA gene in the reproductive tract genomic DNA, diagnostic PCR amplifications were performed using the genus-specific degBurk16SF (newly designed; 5’-TTTTGGACAATGGGSGMAA-3’) and Burk16SR [19] primers. The PCR thermal program consisted of 94°C for 4 min, followed by 30 cycles of 94°C for 30 sec, 60°C for 30 sec, and 68°C for 1 min, and a final extension step at 68°C for 7 minutes. The *Burkholderia* 16S rRNA gene amplicons of all 24 female reproductive tracts produced faint bands on the agarose gel; therefore, additional series of PCR amplifications using degBurk16SF and Burk16SR primers were conducted using the purified universal 1.5-kb 16S rRNA gene amplicons as templates. Purification and sequencing of positive 750-bp amplicons of *Burkholderia* 16S rRNA gene were performed, as described previously [16].

The unidirectional sequencing of 96 clones containing universal 16S rRNA gene amplicons from reproductive tracts of *B*. *insularis* females produced 94 (47 from BiR colony and 47 from BiS colony) clean trace chromatograms. These reads were submitted to the Ribosomal Database Project (RDP) for classification [20]. For each *B*. *insularis* colony (BiR or BiS), the sequences of clones classified as *Burkholderia* were compared with corresponding *Burkholderia* 16S rRNA gene sequences generated from the individual female reproductive-tract DNA preparations and with sequences generated from the corresponding midgut-crypt DNA preparations using multiple sequence alignments of partial 16S rRNA gene reads by MUSCLE 3.7 [21]. Furthermore, the universal 16S rRNA gene amplicons generated from crypt preparations and *Burkholderia*-specific 16S rRNA amplicons of their counterpart reproductive tract preparations that produced clean chromatograms were pairwise aligned using MUSCLE and were trimmed to 705 bp. Forty-six *Burkholderia* 16S rRNA gene sequences and 21 reference sequences were subjected to phylogenetic analyses [16]. *Pandoraea norimbergensis* (GenBank # AF139171) served as the outgroup.

### Fluorescence *in situ* hybridization (FISH)

Dissected midgut crypts and reproductive tracts were placed separately on a pre-cleaned Gold Seal^®^ Fluorescent Antibody RITE-ON microslide (Gold Seal Products, Portsmouth, NH), fixed in 4% paraformaldehyde at 4°C overnight, washed three times in HEPES (10 mM, pH 7.4), and incubated with 0.25 M HCl at room temperature (RT) for 30 minutes. Fixed specimens were dehydrated by sequential incubation in 50, 80, and 100% ethanol (EtOH). Dehydrated specimens, pre-equilibrated with the hybridization buffer (0.1 M Tris-HCl, pH 8.0, 0.9 M NaCl, 0.1% SDS) at 50°C for 15 min, were hybridized with 0.5 μM of 5’-end-labeled oligonucleotide probe Alsym16S [19] at 50°C in darkness for 1 h and rinsed three times in the pre-warmed wash buffer (0.1 M Tris-HCl, pH 8.0, 0.2 M NaCl, 0.1% SDS) at 50°C [22]. Subsequently, washed specimens were chilled in HEPES at 4°C for 2 min, incubated with 1 μg mL^-1^ of the fluorescent stain 4’,6-diamidino-2-phenylindole (DAPI) at RT in darkness for 10 min, washed, and then mounted in the anti-fading agent 1,4-diazabicyclo[2.2.2]octane in glycerol (DABCO). Prepared specimens were examined initially using an epifluorescence microscope (Leitz Laborlux S, Germany) with Texas Red-DAPI filters and photographed. Additional specimens were examined using a laser-scanning confocal microscope (Leica TCS SP5, Germany) with TRITC-DAPI filter set. Hybridization specificity was confirmed using the non-probe control and the Alsym16S probe against the *Escherichia coli* D31 strain. As a positive control, an *in vitro* cultured *B*. *insularis* crypt-derived *Burkholderia* isolate (Bi16MC_R_vitro, ATCC deposit number: TSD-41) [16] was hybridized with the Alsym16S probe.

### Examination of *Burkholderia* in eggs, neonates, and host plants

*Blissus insularis* adults were collected in St. Augustinegrass lawns from multiple locations in Florida, pooled together, and provisioned with surface-sterilized corn cobs as a food source. Two pairs of adults were held separately in clean containers with ventilation to produce eggs, which were collected within 24 h after oviposition using the egg roll [17]. Six eggs from the two pairs were surface-sterilized by a simplified procedure: sequential immersion for 3 min each in 70% EtOH, 5% bleach, and 70% EtOH. An additional seven eggs from the same two pairs were subjected to an additional sterilization procedure: immersion for 30 sec in 0.1% Tween 80 (Fisher Scientific) with vigorous agitation, transferring to 10% acidified bleach (pH 5.5) with a small drop of dish detergent (Ultra Joy^®^, Procter and Gamble, Cincinnati, OH) for 1 min with gentle agitation, and immersion in 70% EtOH for 30 seconds. An additional five unsterilized eggs were examined. Individual eggs were subjected to genomic DNA extraction and diagnostic PCR amplification using the *Burkholderia*-specific 16S rRNA gene primers, as described previously.

In addition to eggs, neonates hatched from both surface-sterilized and control eggs were examined. Five brood replicates, generated from an additional five pairs of *B*. *insularis* adults, were allowed to mate and oviposit, as described previously. Eggs were collected daily and surface-sterilized using the simplified procedure, whereas controls were unsterilized eggs. Subsequently, eggs were placed individually into cells of a clean bioassay tray (BioServe, Frenchtown, NJ) sealed with a perforated tray lid, and held in plastic containers with moistened paper towels at 27 ± 1°C with a 14:10 (L:D) h photoperiod. Eggs were examined daily until neonates hatched. For each brood, hatching rates were recorded. Three to seven neonates from the control and the surface-sterilized group, respectively, were subjected to genomic DNA extraction and diagnostic PCR detection of *Burkholderia* as described above for the eggs.

To investigate the possibility that *B*. *insularis* orally acquires *Burkholderia* from its host plant, three cultivars of St. Augustinegrasses (‘Floratam’, ‘Palmetto’, and ‘Captiva’), which were maintained without exposure to *B*. *insularis*, were used to confirm the presence of *Burkholderia* in St. Augustinegrass. Fresh grass stems (500 mg), free of soil debris, were harvested and homogenized using liquid nitrogen. To determine whether *Burkholderia* was present on the interior and/or exterior of the grass, additional stem preparations of each cultivar were surface-sterilized by immersion for 3 min each in 70% EtOH, 5% bleach, and then 70% EtOH. Grass homogenates were subjected to genomic DNA extraction and to diagnostic PCR detection of *Burkholderia*, as described previously. For each grass cultivar, four sterilized and four unsterilized stem preparations were examined. The seven positive *Burkholderia* 16S rRNA amplicons (~750 bp) (four from Palmetto, three from Floratam) were purified subsequently and subjected to the Sanger sequencing in forward direction to obtain the partial sequence of the *Burkholderia* 16S rRNA gene.

### Rearing of nymphs on plants with cultured *Burkholderia*

Three crypt-derived *Burkholderia* isolates (Bi12MC_S_vitro, Bi16MC_R_vitro, and Bi22MC_R_vitro) [16] were separately cultured in nutrient broth medium, harvested at mid-log exponential phase, and suspended in sterile H_2_O (1× 10^9^ cells mL^-1^). Each *Burkholderia* suspension was spotted onto eggs to ensure that all eggs were evenly coated with bacteria, whereas uncoated eggs were treated with sterile H_2_O. Eggs were subsequently air-dried. Before hatching, a sample of coated eggs and uncoated eggs were examined under a phase-contrast microscope to confirm the presence and absence of bacteria, respectively.

Environmental acquisition of symbionts by *B*. *insularis* was tested in a greenhouse experiment consisting of the following four treatments: 1) neonates that hatched from *Burkholderia*-coated eggs were reared on *Burkholderia*-inoculated plant (chorion^+^/plant^+^); 2) *Burkholderia*-coated eggs were reared on untreated plant (chorion^+^/plant^-^); 3) neonates that hatched from uncoated eggs were reared on *Burkholderia*-inoculated plant (chorion^-^/plant^+^); and 4) neonates that hatched from uncoated eggs were reared on untreated plant (chorion^-^/plant^-^). Overnight cultures of each *Burkholderia* isolate were harvested and suspended in sterile H_2_O (2 × 10^8^ cells mL^-1^). Fifty milliliters of each symbiont suspension were sprayed onto individual Floratam St. Augustinegrass plants that were propagated in pots filled with a mixture of potting soil (Conrad Farfard Inc., Agawam, Massachusetts) and autoclaved fine sand at 1: 1 ratio. Sprayed grasses were air-dried overnight and enclosed in clear plastic cages with ventilation windows on the top and sides (S1 Fig). For each treatment, 20–90 neonates (less than 24 h old) newly hatched from *Burkholderia*-coated/-uncoated eggs were transferred into the cage. The rearing experiment was replicated three times using the *Burkholderia* isolate Bi16MC_R_vitro, replicated twice with the isolate Bi12MC_S_vitro, and conducted once using the isolate Bi22MC_R_vitro.

After 30 d, *B*. *insularis* were removed from the cages to determine their survivorship and respective life stages. To determine whether *B*. *insularis* orally acquired *Burkholderia* from the *Burkholderia*-coated egg surface or from the *Burkholderia*-inoculated plants, randomly selected *B*. *insularis* from each treatment were surface-sterilized, and their midgut crypts were dissected and processed for genomic DNA extraction. Genomic DNA preparations were subjected to BOX-PCR fingerprinting [16]. Bacterial DNA of three cultured *Burkholderia* isolates served as positive controls. An additional crypt-derived isolate (Bi24MC_R_vitro) whose ribotype was clustered with the plant-associated *Burkholderia gladioli* [16] was used as an additional reference. In previous BOX-PCR fingerprinting study [16], the crypt-associated *Burkholderia in vivo* and the cultured counterparts typically had >70% similarity. Therefore, a cut-off (75%) was chosen for the current study to determine if the BOX-PCR patterns from the insect crypts were the same as those from the positive controls. The survivorship and symbiont transmission rate data from three cultured *Burkholderia* isolates were pooled and checked for normal distribution by Kolmogorov-Smirnov test. For the normally distributed survivorship data (N = 24, D = 0.0967, *P* > 0.1500), a one-way ANOVA test (PROC ANOVA, SAS 9.3) was used for treatment comparison. For the non-normally distributed symbiont transmission rate data (N = 24, D = 0.2578, *P* < 0.0100), the Kruskal-Wallis test (PROC NPAR1WAY) was used. A post-hoc test of Dwass, Steel, Critchlow-Fligner (DSCF) was applied for pairwise two-sided multiple comparison analysis (SAS 9.4). Thirty-two insects (12 from Bi16MC_R_vitro, 10 from Bi22MC_R_vitro, 10 from Bi12MC_S_vitro treatment groups), having BOX-PCR profiles that represented approximately 70% of the profiles generated from insect samples fed the untreated plants, were subjected to Sanger sequencing of universal 16S rRNA gene and subsequent phylogenetic analyses, as described previously. Sequences of three inoculated *Burkholderia* isolates and 34 reference sequences were included in phylogenetic analyses.

### Nucleotide sequence accession numbers

All DNA sequences obtained from this study were deposited in the GenBank nucleotide sequence databases with these accession numbers: KP683095 to KP683116, KP713811 to KP713834, KX232109 to KX232140, and KX233264 to KX233270.

## Results

### *In situ* detection of *Burkholderia* in midgut crypts and reproductive tracts of *B*. *insularis*

**Fig 1:**
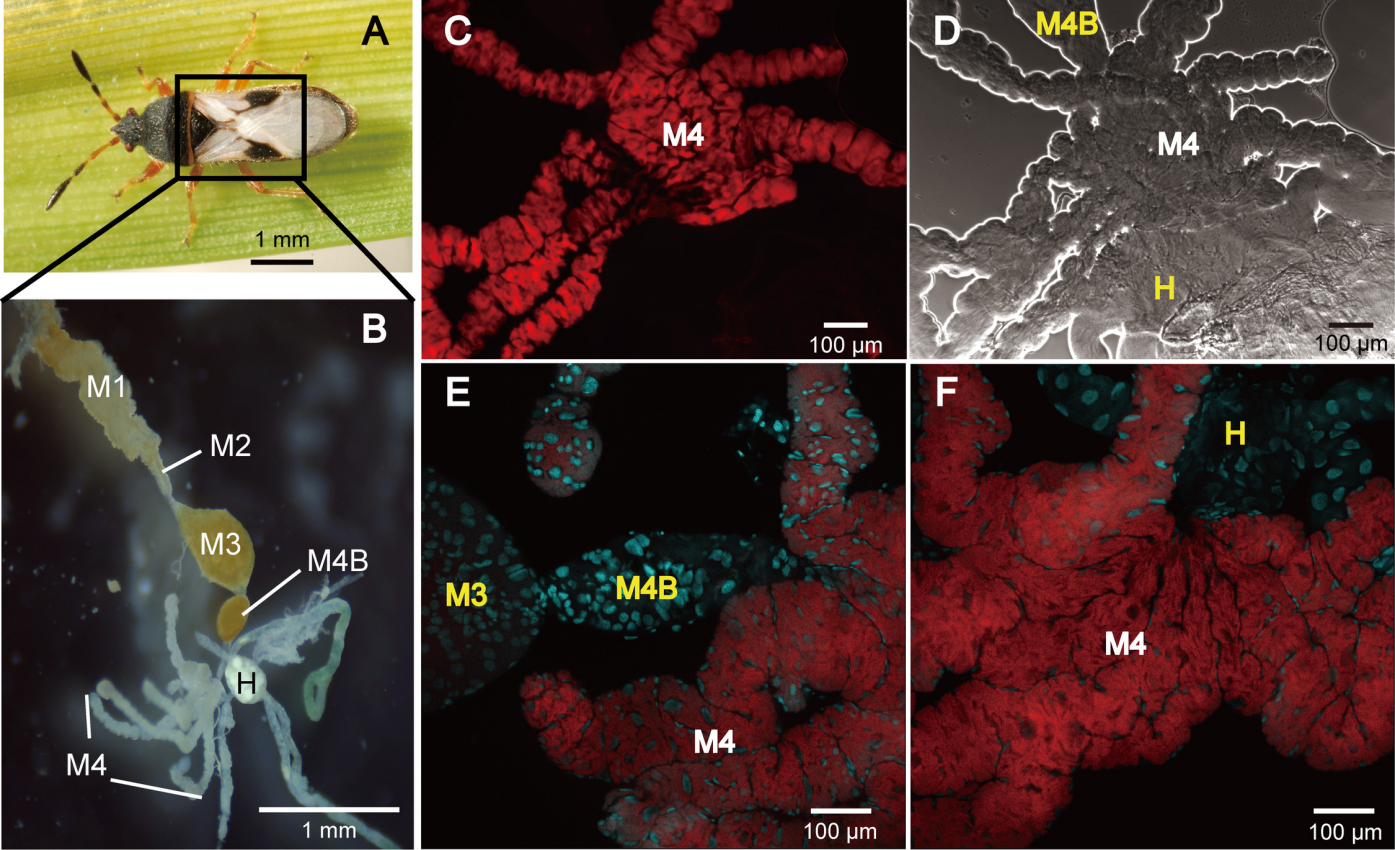
Visualization of *Burkholderia* in the digestive tract of *Blissus insularis*. (A) An adult *B*. *insularis*. (B) Dissected digestive tract. (C) Epifluorescence micrograph of FISH targeting *Burkholderia* 16S rRNA gene (red) in M4. (D) Phase contrast micrograph of the same areas. (E-F) Focus stacked laser scanning confocal micrographs of FISH localization of *Burkholderia* (red) in M4. Cyan signals indicate host nuclei stained with DAPI. Abbreviations: M3, midgut third section; M4, midgut fourth section with crypts; M4B, M4 bulb; H, hindgut. The labeling corresponds to that used for *Cavelerius saccharivorus* [12].

**Fig 2:**
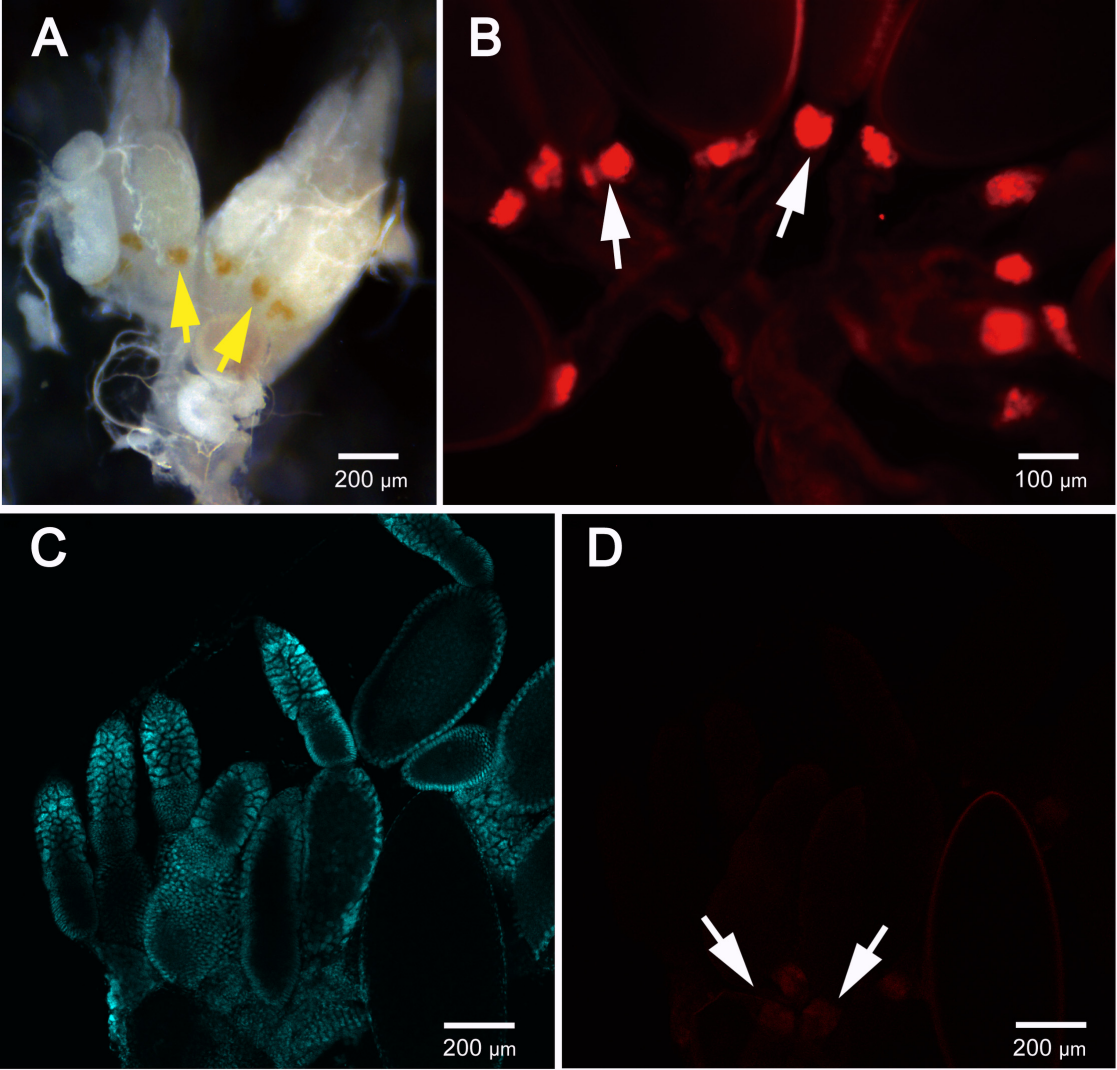
Micrograph of *Blissus insularis* female reproductive tracts. (A) Dissected female reproductive tracts of *B*. *insularis* with pedicel areas in yellow color (arrows) within ovarioles. (B) Epifluorescence micrograph of reproductive tracts without FISH, indicating the false signal by arrows, possibly caused by the melanin being deposited in the pedicel area. (C-D) Laser scanning confocal micrographs of reproductive tracts stained with DAPI and FISH.

FISH analyses using both epifluorescence and confocal microscopy revealed that *Burkholderia* bacteria colonized the midgut crypts rather than other regions of the digestive tract (*i*.*e*., anterior midgut, hindgut) of *B*. *insularis* adults (Fig 1). Dense populations of *Burkholderia* were confined to the lumen of crypts, supporting the previous findings that the midgut crypts are the specialized symbiotic organ in *B*. *insularis* [15,16]. Both female and male reproductive tracts were subjected to FISH, but no detectable *Burkholderia* signal occurred in either one. Initially, a red signal, forming a red halo, was detected in the pedicel area of each ovariole during FISH analyses using the epifluorescence microscope fitted with Texas Red filter. However, negative controls without the FISH probe confirmed that this was a false positive signal (Fig 2B), likely caused by the melanin deposited in the pedicel area (Fig 2A). Re-examination of additional samples using the confocal microscope with the more specific TRITC filter confirmed that it was a false positive signal (Fig 2C and 2D). The FISH probe readily hybridized to the cultured *Burkholderia* (Bi16MC_R_vitro), but it failed to produce positive signals in the no-probe and *E*. *coli* controls (S2 Fig), confirming specificity of the hybridization.

### Bacterial ribotypes in midgut crypts and reproductive tracts of *B*. *insularis*

Ribotying-based identification of bacteria inhabiting midgut crypts and reproductive tracts of *B*. *insularis* female individuals revealed a single *Burkholderia* ribotype in their respective crypts and a mixed bacterial community with low titers of *Burkholderia* in their reproductive tracts. Twenty-two of the 24 examined crypt-associated 16S rRNA gene amplicons (~1.4 kb) produced chromatograms free of mixed reads within the target sequence and were identified as belonging to the genus *Burkholderia*. The remaining two (Bi10MC_R and Bi02MC_S) crypt-associated amplicons and all 24 examined reproductive tract-associated 16S rRNA gene amplicons produced mixed chromatograms. PCR reactions of reproductive tract genomic DNA using *Burkholderia*-specific primers produced faint bands corresponding to the target 750-bp *Burkholderia* 16S rRNA gene amplicons on agarose gels (S3 Fig). Subsequent PCR amplifications with *Burkholderia*-specific primers conducted on the universal 16S rRNA amplicon preparations detected positive *Burkholderia* 16S rRNA amplicons in the 24 female reproductive tracts (S3 Fig) and in the two ‘mixed’ crypt reads. Sanger sequencing of the re-amplified *Burkholderia* 16S rRNA gene sequences from 23 female reproductive-tract preparations and one crypt (Bi10MC_R) DNA preparation produced chromatograms free of mixed reads. The mixed reads (Bi09RT_S and Bi02MC_S) in re-amplified *Burkholderia* 16S rRNA reads were excluded from the phylogenetic analysis.

Phylogenetic analyses placed the 16S rRNA gene partial sequences of crypt genomic DNA preparations and those of their corresponding reproductive tracts within five major clades, the SBE, the PBE, the BCC, the pathogen (PC), and the soil (SC) clades (S4 Fig). Half of the 46 sequences affiliated with the SBE clade (bootstrap value, 73%) that were identified previously in *B*. *insularis* [16]. Twelve sequences were placed in two previously defined clades: seven sequences (15%) in the PBE (bootstrap value, 79%) and five sequences (11%) in the BCC clade (bootstrap value, 63%). In addition, eight sequences (18%) were clustered in clade PC with *B*. *gladioli*-associated strains (bootstrap value, 98%) that were placed within the BCC clade in the previous report [16]. Two sequences (4%) were grouped in clade SC with soil-derived *Burkholderia* (bootstrap value, 91%). However, one sequence (Bi10MC_R, 2%) was not affiliated with any clades.

Of 22 female *B*. *insularis* examined, seven females (five from BiR colony and two from BiS colony) harbored *Burkholderia* in crypts having ribotypes identical to those in their respective reproductive tracts (S1 Table). For the other 15 individuals, eight females (four from BiR and four from BiS) had similar 16S sequences with ≤ 3% of nucleotide difference, whereas seven females (four from BiR and three from BiS) harbored *Burkholderia* ribotypes with > 3% of nucleotide difference between aligned crypt and reproductive tract *Burkholderia* 16S rRNA gene partial sequences (~700 bp). The SNPs identified in these pairwise alignments were localized in the hypervariable V3-V7 regions [16].

Analyses of the partial 16S rRNA gene sequences (~900 bp) identified the 47 clones from the BiR-pool to Grammaproteobacteria (n = 34), Betaproteobacteria (n = 12), and Alphaproteobacteria (n = 1) (S2 Table). All twelve betaproteobacterial clones were identified as members of the genus *Burkholderia*. Among them, three were identical to the Bi02RT_R and Bi10RT_R sequences identified by PCR amplification of the uncloned BiR reproductive-tract DNA preparations. The other nine clone sequences displayed a best match (97–99% similarity) to Bi02RT_R, Bi07RT_R, or Bi10RT_R sequence. None of the clones from the BiR pool matched the fourth pooled sample (Bi11RT_R) sequence. Similarly, the 47 clones derived from the pooled 16S rRNA amplicons of the BiS female reproductive tracts contained Gammaproteobacteria (n = 35), Betaproteobacteria (n = 11), and Bacteroidetes (n = 1) (S2 Table). All eleven betaproteobacterial clones were identified as members of the genus *Burkholderia*. Four *Burkholderia* clones had identical sequences to the Bi01RT_S and Bi10RT_S, whereas the other seven clones had 99% sequence similarity to the sequences of Bi10RT_S and Bi11RT_S. No clone sequences matched to the fourth pooled Bi08RT_S. Comparison of these cloned 16S rRNA sequences to the sequences generated from corresponding BiR and BiS *B*. *insularis* midgut crypts showed that most *Burkholderia* clone sequences generated from reproductive-tract DNA preparations also had 97–100% similarity to the sequences of their respective midgut crypts, except one clone in the BiR pool that had 96% sequence similarity to its midgut crypt preparation (Bi07MC_R) (S2 Table).

### Examination of *Burkholderia* in eggs, neonates, and host plants

Diagnostic PCR amplifications of the *Burkholderia*-specific 16S rRNA gene revealed that *Burkholderia* were undetectable in all 18 examined eggs, regardless of the surface-sterilization procedure (S5A Fig). Similarly, diagnostic PCR failed to amplify the detectable *Burkholderia* 16S rRNA gene amplicon targets in DNA preparations of 24 and 25 *B*. *insularis* neonates hatched from unsterilized and surface-sterilized eggs, respectively (S5B Fig). However, the midgut crypts of their respective *B*. *insularis* parents harbored high titers of *Burkholderia*. Weak but detectable *Burkholderia* signals were also found in their parental reproductive tracts. Detection of *Burkholderia* in the symbiotic organ (midgut crypts) and reproductive tracts of parents rather than their newly born offspring suggested the gut symbiont *Burkholderia* were not acquired prenatally or via symbiont-contaminated egg surface.

Faint PCR amplicons at the target size (~750 bp) were detected in all three examined St. Augustinegrass cultivars, regardless of the surface sterilization: 88% in Palmetto, 100% in Floratam, and 100% in Captiva. In the unsterilized samples, the PCR amplicons were more intense than those from the surface-sterilized samples (S6 Fig). Seven partial sequences (522 bp) of *Burkholderia* 16S rRNA gene from the unsterilized rearing-used St. Augustinegrass samples revealed that, regardless of the grass cultivar, they had 99–100% sequence similarity (0 to 1 SNP) to each other and identified as *Burkholderia*, closely related to the *B*. *gladioli* strain (accession number: DQ355168) and other crypt-associated *Burkholderia* in *B*. *insularis* within the BCC clade.

### Rearing of nymphs on plants with cultured *Burkholderia*

Phase-contrast microscopy of *Burkholderia*-coated eggs revealed the presence of numerous bacteria on the chorion surface, whereas no bacteria were detected on uncoated egg surfaces (S7 Fig). A total of 1,385 *B*. *insularis* neonates emerging from coated and uncoated eggs were subjected to the infection experiment on live plants, using three *Burkholderia* isolates. After one month of rearing on live plants, regardless of the *Burkholderia* inoculum source, approximately 50% of the neonates developed into older nymphs (fourth and fifth instars) or adults. For the 702 neonates that were reared on the *Burkholderia*-inoculated plants, 42% from the egg chorion-coated and 43% from the uncoated neonates had survived at the termination of the experiment. Even though neonates were reared on the untreated plants, survivorship of both chorion-coated (65%) and the uncoated eggs (51%) were similar to those from the *Burkholderia*-inoculated plants (Table 1). No difference in survivorship was found among four treatments (*F* = 1.0300, df = 3, *P* = 0.4021).

**Table 1:** The survivorship and symbiont transmission rate of *Blissus insularis* reared on the live plant inoculated with *Burkholderia* isolates.

	% Survivorship (no. survivor/total)^*a*^				% Symbiont transmission (no. positive/total)^*b*^			
Treatment	Bi12MC_S	Bi16MC_R	Bi25MC_R	Total^*c*^	Bi12MC_S	Bi16MC_R	Bi25MC_R	Total
chorion^+^/plant^+^	43 (63/145)	55 (66/119)	23 (23/99)	42 (152/363)	75 (15/20)	78 (18/23)	100 (9/9)	81 (42/52) a
chorion^-^/plant^+^	42 (63/149)	34 (38/112)	59 (46/78)	43 (147/339)	60 (12/20)	87 (20/23)	70 (7/10)	74 (39/53) a
chorion^+^/plant^-^	69 (102/147)	65 (78/120)	59 (51/86)	65 (231/353)	30 (6/20)	7 (2/30)	10 (1/10)	15 (9/60) b
chorion^-^/plant^-^	46 (75/163)	76 (79/104)	24 (15/63)	51 (169/330)	0 (0/20)	0 (0/29)	0 (0/10)	0 (0/59) c
			*F* (df)^*d*^	1.0300 (3)			*χ*^2^ (df)^*e*^	19.1870 (3)
			*P*	0.4021			*P*	0.0003

^*a*^ After 30-d rearing, the number of surviving *B*. *insularis* divided by the total number of neonates that were subjected to each treatment.

^*b*^ The number of examined *B*. *insularis* that exhibited positive symbiont transmission divided by the total number of *B*. *insularis* that were subjected to BOX-PCR analyses.

^*c*^ Data from three cultured *Burkholderia* isolates were pooled for each treatment.

^*d*^ *F* value and degree of freedom, analyzed by one-way ANOVA test.

^*e*^ Values of Chi-square and degree of freedom, analyzed by Kruskal-Wallis test. Different letters in the column indicate statistically significant difference between each treatment (Dwass, Steel, Critchlow-Fligner Method; *P* < 0.05).

A total of 224 *B*. *insularis* of mixed ages and sexes were subjected to crypt dissection, genomic DNA extraction, and BOX-PCR fingerprinting. Regardless of the ribotype of inoculated *Burkholderia* isolate, 81% of *B*. *insularis* that were double-infected with cultured *Burkholderia* (chorion^+^/plant^+^) had the same BOX-PCR patterns as the inoculated *Burkholderia* isolate, suggesting that these insects successfully acquired cultured *Burkholderia* from the environment (via contaminated egg surface and/or plant). When cultured *Burkholderia* were applied only to live plants, *B*. *insularis* neonates generated from uncoated eggs (chorion^-^/plant^+^) also exhibited 73% of symbiont transmission rates. For those *B*. *insularis* reared on the untreated plants, only 15% of the egg chorion-coated insects (chorion^+^/plant^-^) acquired cultured *Burkholderia*, whereas none of the negative controls (chorion^-^/plant^-^) had the same BOX-PCR patterns as the inoculated *Burkholderia* isolate (S8 Fig). Significantly higher symbiont transmission rates were observed in the *B*. *insularis* that were reared on *Burkholderia*-inoculated plants, relative to the offspring from coated eggs reared on untreated plants (*χ*^2^ = 19.1870, df = 3, *P* = 0.0003) (Table 1). These results demonstrate that the cultured *Burkholderia*, regardless of its ribotype, was acquired by *B*. *insularis* neonates from the coated egg surface and/or the environment (*i*.*e*., plants and soils). *Blissus insularis* from the untreated plant cages harbored diverse *Burkholderia* with ribotypes that were distinct from those of the three inoculated *Burkholderia* isolates.

**Fig 3:**
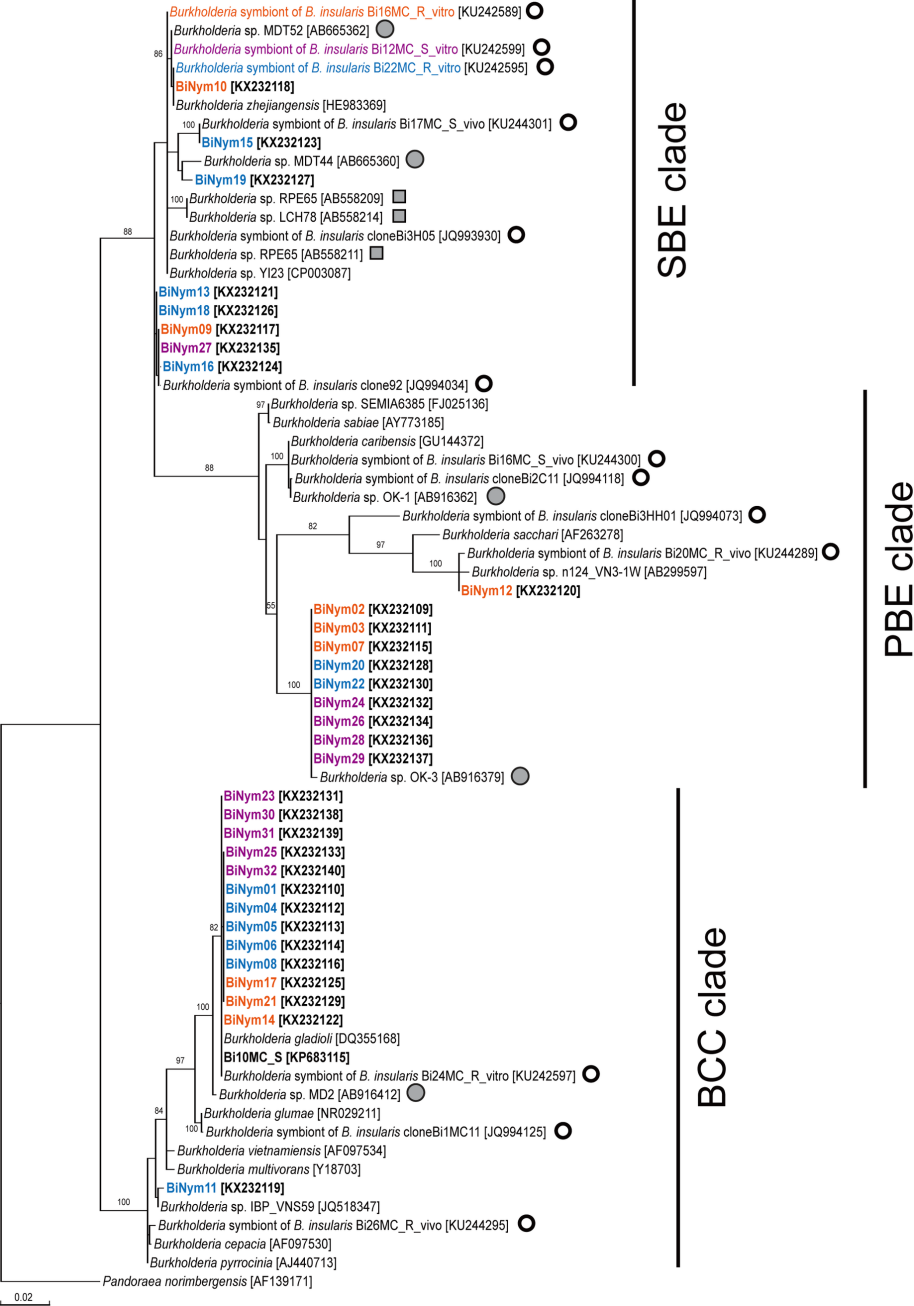
Phylogenetic relationship of *Burkholderia* obtained from *Blissus insularis* nymph midgut crypts. 16S rRNA gene sequences (1.3 kb) obtained in the current study are shown in bold. Numbers at the tree nodes represent the maximum-likelihood bootstrap values obtained after 100 repetitions; only values over 50 are shown. In brackets are shown nucleotide sequence accession numbers in the GenBank. Clear and gray circles denote the *Burkholderia* isolates detected previously in the *B*. *insularis* [15,16] and in *C*. *saccharivorus* [12], respectively; squares denote the *Burkholderia* isolates detected in other heteropteran hosts. The sequences of nymph midgut crypts (denoted as BiNym01 to BiNym32) derived from the same treatment are highlighted by same colors. The clades SBE, PBE, and BCC correspond to those described in references [12], [23], and [24], respectively.

The universal 16S rRNA gene sequences of 32 examined crypt preparations from insects that fed on the untreated plants produced clean trace chromatograms. Based on the partial sequences (~1.3 kb), these ribotypes belonged to *Burkholderia* and were grouped into the previously reported SBE, PBE, and BCC clades [16] (Fig 3). Eight sequences (25%) were grouped within the SBE clade, to which the three inoculated *Burkholderia* isolates belonged; whereas the remaining 24 were affiliated with *Burkholderia* strains in other two clades: 31% in the PBE clade and 44% in the BCC clade.

## Discussion

*Blissus insularis* harbors high densities of exocellular *Burkholderia* symbionts in the lumen of specialized midgut region, called crypts [15]. A recent study [16] revealed that the majority of crypts from individual *B*. *insularis* contained a single ribotype of this symbiont. In the current study, a low-density bacterial community was detected in the female reproductive tracts of *B*. *insularis* individuals from both BiR and BiS laboratory colonies. In addition, direct PCR amplification and sequencing of 16S rRNA gene clones revealed low titers of *Burkholderia* in these reproductive tracts.

The *Burkholderia* sequences detected in the recombinant clones matched six of the eight sequences of the gene-specific amplicons comprising the pooled reproductive-tract DNA preparations. The inability to detect the other two sequences may be due to differences in titer, cloning bias, or the limited sequencing of the clones. In addition to *Burkholderia*, non-*Burkholderia* sequences also were found in *B*. *insularis* female reproductive tracts and were related to Gammaproteobacteria (*Escherichia*, *Morganella*, *Serratia*, Pseudomonadaceae, and Xanthomonadaceae), Alphaproteobacteria (*Sphingomonas*), and Bacterioidetes (*Myroides*) (see S2 Table). Previous assays have shown that some BiR and BiS individuals also harbor PCR-detectable *Wolbachia* 16S rRNA gene signals in the female reproductive-tract DNA preparations [25]. *Wolbachia* is an endocellular bacterium known to infect and impact the reproductive fitness of many insects [26–28], however, its role in *Blissus* is presently unknown.

Results of high-throughput sequencing have demonstrated that reproductive tracts of insects may harbor a complex microbiome. For example, the female reproductive tracts of the mosquito *Anopheles* [29] and fruit fly *Bactrocera* [30] contain hundreds of species-level sequence clusters distributed over numerous bacterial phyla. Although the roles of endosymbiotic *Wolbachia* in host biology have been deciphered in many insects [26,31,32], little is known about the function of other microbial components in reproductive tissues. The limited number of bacterial taxa detected in *Blissus* as compared to both mosquitoes [29] and fruit flies [30] is due in part to the limitations associated with sequencing full-length 16S rRNA gene clonal libraries as compared to the direct high-throughput sequencing approach.

A high degree of similarity (97–100%) of the 16S rRNA gene sequences between most (15 out of 22) female reproductive tracts and their respective midgut crypts suggests that *B*. *insularis* female individuals may harbor the same *Burkholderia* ribotype in reproductive tracts and in crypts. How the gut-associated exocellular bacteria in *Blissus* relocate to the female reproductive tract is unknown. The inability of FISH to detect a *Burkholderia* signal in the reproductive tracts may be due to the low titers of the target rRNA molecules in host tissues [33]. Alternatively, the lack of a FISH signal may imply that the PCR detection of *Burkholderia* in female reproductive tracts, successful only with re-amplification of PCR products, represents either an undetectable titer or contaminant. Importantly, other findings, including negative PCR results of egg and neonate preparations, failed to provide evidence for vertical (transovarial or postnatal) transmission of *Burkholderia* in *B*. *insularis*. This assumption needs to be validated with a different approach, such as the hypersensitive VNTR (Variable-Number-Tandem-Repeat) fingerprinting strategy used to detect low titer infections of *Wolbachia* in tsetse flies [34]. Nevertheless, for exocellular gut symbionts that are maternally transmitted in insects, no evidence has shown that these symbionts are transovarially transmitted [3,4] despite the PCR-based detection of symbionts in ovarioles [35].

Phylogenetic analyses of sequences derived from crypt DNA preparations of *B*. *insularis* females revealed ribotypes that belong to two additional *Burkholderia* groups (PC and SC clades, S4 Fig). The eight sequences grouped in the PC clade in the current study were closely associated with three strains (*B*. *gladioli*, and two *C*. *saccharivorus*-associated *Burkholderia*), which were placed within the BCC clade in the previous report [16]. This may reflect a difference in length between 16S rRNA gene sequences (705bp) subjected to the phylogenetic analyses in the current study and those (~1.4 kb) in the previous study [16], rather than the a new group of *Burkholderia*. However, two partial 16S rRNA gene sequences generated from crypts grouped in a clade (SC clade) not previously documented in Blissidae [12,16]. Similar to the midgut crypts, the reproductive tracts also contained diverse *Burkholderia* ribotypes ground in multiple clades (see S4 Fig). Some hemipterans, such as acanthosomatid [2], plataspid [5], and lygaeid bugs [36,37], contain gut- [2,5] or gonad-associated [36,37] symbionts, which are vertically transmitted and form coherent monophyletic groups aligned with the phylogenies of their respective host insects. On contrary, other bugs in the Lygaeoidea and Coreoidea superfamilies that acquire gut symbionts via the environment (*i*.*e*., soils and plants) [11] display discrepancies between symbionts and host phylogenies [38]. The later situation probably mirrors the promiscuous host-symbiont relationship in *B*. *insularis* [15,16]. In addition to phylogenetic association, the symbiont genome size also may reflect its transmission route [39]. Specifically, vertically transmitted gut symbionts of acanthosomatid [2] and plataspid [2] have relatively small genomes (0.7 to 0.9 Mb) compared to the environmentally acquired gut symbiont *Burkholderia* in *Riptortus pedestris* (7.0 to 8.7 Mb) [40,41]. Similar to *R*. *pedestris*, *B*. *insularis* also harbors *Burkholderia* with relatively large genomes (6.6 to 8.7 Mb) [16] in midgut crypts. These findings further suggest that the vertical transmission of *Burkholderia* in *B*. *insularis* remains controversial.

Many heteropterans have evolved postnatal transmission mechanisms to ensure the acquisition of essential gut symbionts by offspring, including: 1) the probing of symbiont-contaminated egg chorion as shown in the families Acanthosomatidae and Pentatomidae [2–4,42]; 2) deposition of the symbiont-containing capsules on the underside of egg masses by females in the Plataspidae [5,6]; 3) excretion of the symbiont-containing feces by infected adults in the Parastrachiidae and Reduviidae [7–9]; 4) and/or acquisition from the environment where the free-living symbionts acquired by members of the Alydidae [11]. Most gut symbionts that are transmitted via the first three routes belong to Gammaproteobacteria [3–5,9] and Actinobacteria [8], whereas the environmentally-acquired gut symbiont in alydids belongs to the genus *Burkholderia* in the Betaproteobacteria [11,19].

In *B*. *insularis*, *Burkholderia* is the dominant bacteria inhabiting the midgut crypts, and it appears to play an important role in maintaining host fitness [15]. Even though low titers of *Burkholderia* were detected by qPCR amplifications in field-collected *B*. *insularis* eggs [15], data from the current study failed to confirm the proposed vertical transmission by the previous study [15]. In stinkbugs (acanthosomatids and pentatomids), the newly hatched neonates typically aggregate on eggs and probe the symbiont-containing chorion for one to six hours to acquire symbionts that were deposited by females during oviposition [2–4]. Diagnostic PCR has confirmed that stinkbug eggs contained symbionts after oviposition, that nymphs allowed to probe the chorion acquired the symbiont, and that the nymphs removed immediately from chorion after hatching are symbiont-free [4]. The egg-chorion-probing behavior displayed in stinkbugs has not been observed in *B*. *insularis* (personal observation). Importantly, diagnostic PCR amplifications of the current study failed to detect *Burkholderia* in unsterilized eggs and in neonates of *B*. *insularis*. Second, the symbiont-containing capsules that are deposited by the female plataspid stinkbug are attached to the egg masses, arranging in two parallel lines. Upon hatching, plataspid neonates constantly probe the capsule to acquire symbionts [6]. However, no such attached capsule is observed in *B*. *insularis* [15]. Unlike egg masses laid in strings or in clusters by plataspids and pentatomids [4,6], *B*. *insularis* eggs are deposited singly and randomly between grass blades [43]. This oviposition strategy also suggests that *B*. *insularis* females are unable to transmit the gut symbiont through symbiont-containing mucous secretion, which is a specialized maternal transmission route in the subsocial parastrachiids [9]. Specifically, in these insects, the mother guards the egg masses in a cluster after oviposition, and she excretes the obligate-symbiont-containing mucus from the anus onto eggs several minutes before they hatch. Neonates then acquire the symbiont after hatching [9]. Diagnostic PCR amplification of fecal bacteria from *B*. *insularis* adults contained detectable *Burkholderia* 16S rRNA gene signals [25]. These findings suggest the possibility that *B*. *insularis* neonates may acquire *Burkholderia* through the symbiont-containing feces deposited by the infected nymphs and adults onto the plant substrate, as demonstrated in the blood-feeding reduviids [8]. Typically, *B*. *insularis* forms dense and multigenerational aggregations while they feed on St. Augustinegrass [44]. This behavior possibly contributes to contact to feces, which may maintain the complex of *Burkholderia* detected in both field-collected populations [15] and in laboratory-maintained colonies of *B*. *insularis* [16].

Other laboratory studies have reported that *B*. *insularis* neonates reared solely on corn [17] or on freshly clipped sections of St. Augustinegrass [13] displayed high levels of mortality at early instars. In addition to the cut grass assays, attempts to rear neonates on *Burkholderia*-loaded corn juice food failed [25], suggesting the importance of live grass as a food source during the early instars. This requirement constrained our ability to maintain axenic insects and to conduct experiments such as those performed on the seed-feeding *Riptortus*, which is able to feed and develop on sterilized soybean seeds in the absence of symbiont *Burkholderia* [11]. Therefore, a rearing experiment using live plants was applied in the current study.

Significantly, diagnostic PCR of host plant DNA confirmed the presence of *Burkholderia*; the ribotypes of these sequences were grouped with the *B*. *gladioli* strain. These findings indicate that the host plant may serve as a direct source of gut symbiont *Burkholderia* for *B*. *insularis* that feed by piercing through the innermost leaf sheath [14]. Plant-to-insect transmission is supported also by the rearing experiment using live St. Augustinegrass plants, showing that survivorship of *B*. *insularis* in the negative control group (chorion^-^/plant^-^) were similar to those in the single- (chorion^+^/plant^-^; chorion^-^/plant^+^) and double-infected groups (chorion^+^/plant^+^). In other insect-bacterial symbiosis, the horizontal transmission of endocellular bacteria *Cardinium* between different sap-feeding insect species is mediated by the host plant [45]. Symbiont bacteria, which were released from the salivary glands of the leafhopper *Scaphoideus titanus* during feeding, entered into the plant tissues and were subsequently detected in midguts of other phloem feeders exposed to the same plant [45]. Although speculated, the phloem tissue of St. Augustinegrass may serve as a reservoir of the symbiont *Burkholderia* for *B*. *insularis* facilitating acquisition of this bacterium by offspring. Additionally, the low rate of transmission (15%) from eggs containing a large bacterial load to the offspring suggests that, at best, the egg surface is a potential minor source for oral acquisition of symbionts. In a related blissid species, *C*. *saccharivorus*, the gut symbiont *Burkholderia* is acquired primarily from the environment, although the vertical transmission via egg surface contamination coexists in 30% of examined nymphs [12].

In this study, detection of *Burkholderia* in St. Augustinegrass that is related to plant-associated *Burkholderia* strains suggests that St. Augustinegrass can harbor these bacteria. Through feeding, *B*. *insularis* may initially acquire endophytic *Burkholderia* from grass that serves as an alternative host for *Burkholderia* (see prior section). Aggregated *B*. *insularis* nymphs and adults typically feed on grass phloem sap [44] and may elicit horizontal transmission by releasing gut-symbiotic *Burkholderia* into the wounded area through fecal materials or regurgitation. It should be noted that several *Burkholderia* are known for their pathogenicity in plant disease, including *B*. *gladioli*, *B*. *glathei*, and *B*. *glumae* [23,46]. These phytopathogenic bacteria typically have a broad host plant range and induce leaf browning and wilting [46]. Currently, the mechanism of the *B*. *insularis* feeding-induced grass damage [13,14,47] is not fully understood. Early studies suggested that *Blissus* species damage host plants by the withdrawal of phloem accompanied by the blockage of the plant’s conductive tissues, the result of insect salivary sheaths produced during feeding [14]. The potential role of *B*. *insularis*-associated *Burkholderia* in St. Augustinegrass damage remains unknown and is of great interest to investigate further.

In summary, a series of experiments demonstrates that the primary gut symbiont transmission route in *B*. *insularis* is oral acquisition from sources in the environment (plants and soils). The vertical transmission via symbiont-contaminated egg surface upon oviposition found in *C*. *saccharivorus* [12], however, is less likely as a major route for acquiring gut-symbiotic *Burkholderia* in *B*. *insularis*. Typically, environmentally transmitted symbiosis involves inter-partner recognition and selection between symbionts and hosts and requires delicate molecular cross-talk to accomplish selective colonization [48]. Further investigation of the symbiotic factors (acquisition, accommodation, and persistence) [49] that affect the symbiont *Burkholderia* association with the host *B*. *insularis* is needed.

## Supporting Information

S1 Fig Rearing of *Blissus insularis* nymphs on plants with cultured *Burkholderia*. (A) Unhatched eggs and one newly hatched neonate that was transferred into the cage and was reared on plants with/without cultured *Burkholderia*. (B) Plastic cage built for rearing *B*. *insularis* on live St. Augustinegrass to examine the symbiont transmission route.
(TIF)

S2 Fig The specificity of FISH probe. Phase contrast and epifluorescence (FISH of the same areas) micrographs confirm the specificity of FISH probe (Alsym16S) against the culturable *Burkholderia* Bi16MC_R_vitro isolate from crypts of *Blissus insularis*, but not against *E*. *coli* or the non-probe control. Scale bars = 10 μm.
(TIF)

S3 Fig The PCR gels of 16S rRNA genes detected in *Blissus insularis* reproductive tracts. Initial PCR amplifications of the universal 16S rRNA gene (~1.5 kb) (A) and the *Burkholderia* 16S rRNA gene (~750 bp) (B) detected in the genomic DNA of reproductive tracts from different females. Lanes in panels A and B: 1–5 = Bi09RT_R to Bi13RT_R; 6–8 = Bi09RT_S to Bi11RT_S; 9 = non-template control. The arrows in panel B indicate the faint bands of target *Burkholderia* 16S rRNA amplicons. (C) The subsequent reamplifications of *Burkholderia* 16S rRNA gene in the purified universal 16S rRNA amplicons detected in all examined reproductive tract samples. Lanes in panel C: 1–5 = Bi09RT_R to Bi13RT_R; 6–8 = Bi09RT_S to Bi11RT_S; 9 = Bi10MC_R served as a positive control; 10 = non-template control. Standard markers are HyperLadder^™^ II (Bioline, Taunton, MA).
(TIF)

S4 Fig Phylogenetic relationship of *Burkholderia* obtained from *Blissus insularis* females. (DOCX)

S5 Fig Diagnostic *Burkholderia*-specific PCR analyses of *Blissus insularis* eggs and neonates. (A) The initial PCR amplifications of the *Burkholderia* 16S rRNA gene (~750 bp) did not detect *Burkholderia* in the genomic DNA of eggs, which were from two pairs of *B*. *insularis* adults, regardless of the surface sterilization treatment. Lanes 1–5 = five eggs that were not surface-sterilized; 6–11 = six eggs that were sterilized using a simplified method (see detail in Materials and Methods section); 12–18 = seven eggs that were sterilized using a complicated method; 19 = crypt genomic DNA of a female parent; 20 = reproductive tract genomic DNA of a female parent; 21 = non-template control. (B) The initial PCR amplifications of the *Burkholderia* 16S rRNA gene did not detect *Burkholderia* in the genomic DNA of neonates (less than 24-hour old), which were from five pairs of *B*. *insularis* adults, regardless of the egg surface sterilization treatment. Lanes 1–10 = ten neonates that were hatched from not surface-sterilized eggs; 11 = crypt genomic DNA of a female parent; 12 = reproductive tract genomic DNA of a female parent; 13–23 = eleven neonates that were hatched from sterilized eggs; 24 = non-template control. Standard markers are HyperLadder^™^ I (Bioline, Taunton, MA).
(TIF)

S6 Fig The PCR gel of *Burkholderia* 16S rRNA gene detected in St. Augustinegrass. The initial PCR amplifications of *Burkholderia* 16S rRNA gene detected target amplicons (~750 bp; indicated by arrows) in the genomic DNA from St. Augustinegrass stems of three cultivars. Lanes: 1–2 = donated ‘Floratam’; 3–4 = donated ‘Palmetto’; 5–6 = donated ‘Captiva’; 7–9 = rearing-used ‘Floratam’; 10 = non-template control. Standard marker is HyperLadder^™^ I (Bioline, Taunton, MA). NS, non-surface-sterilized; S, surface-sterilized.
(TIF)

S7 Fig The phase-contrast microscopy of *Burkholderia*-coated and uncoated egg chorions. The *Burkholderia*-coated and uncoated egg chorions were left over by the newly hatched *Blissus insularis* neonates, which were used in the rearing of neonates on live plants with cultured symbionts experiment. In the *Burkholderia*-coated treatment, the presence of rod-shaped bacteria on the surface of egg chorion was observed after 5-day of application of *Burkholderia* suspension (1× 10^9^ cells mL^-1^). No bacterium was found in the control group.
(TIF)

S8 Fig The representative BOX-PCR gel of crypt-associated bacteria *in vivo* from *Blissus insularis*. Lanes 1–34 indicate the *B*. *insularis* reared on live plants with or without cultured *Burkholderia* inoculation. Abbreviations: P = the positive control (the inoculated *Burkholderia* isolate, Bi16MC_R_vitro); a = the treatment chorion^+^/plant^+^ (see text for details); b = the treatment chorion^-^/plant^+^; c = the treatment chorion^+^/plant^-^; d = treatment chorion^-^/plant^-^. R = the reference cultured *Burkholderia* isolate (Bi24MC_R_vitro). NTC = non-template control. Standard markers are HyperLadder^™^ I. Star indicates the lane-based samples that had ≥75% similarity with the positive control (P).
(TIF)

S1 Table The single-nucleotide polymorphisms detected in *Blissus insularis*. (DOCX)

S2 Table Putative phylogenetic affiliation of the 16S rRNA gene clones obtained from the reproductive tracts of four BiR and four BiS *Blissus insularis* females. (DOCX)
