# Supplementary figures and images for: Environmental Transmission of the Gut Symbiont Burkholderia to Phloem-Feeding Blissus insularis
Source: PLoS One. 2016 Aug 22;11(8):e0161699. doi: 10.1371/journal.pone.0161699 (PMC4993365; doi:10.1371/journal.pone.0161699)

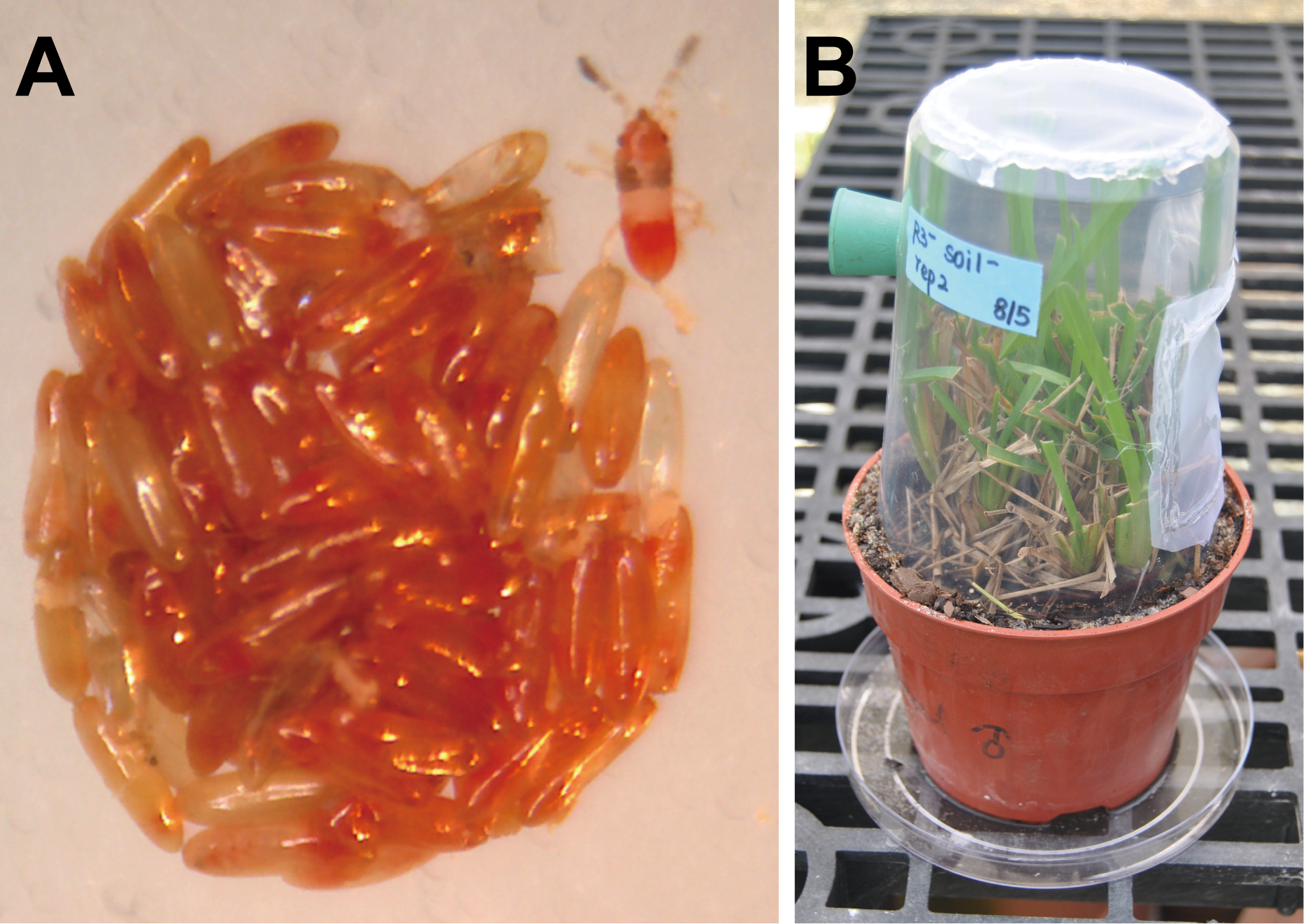

Supplement: S1 Fig — (A) Unhatched eggs and one newly hatched neonate that was transferred into the cage and was reared on plants with/without cultured Burkholderia. (B) Plastic cage built for rearing B. insularis on live St. Augustinegrass to examine the symbiont transmission route. (TIF) [file pone.0161699.s001.tif]

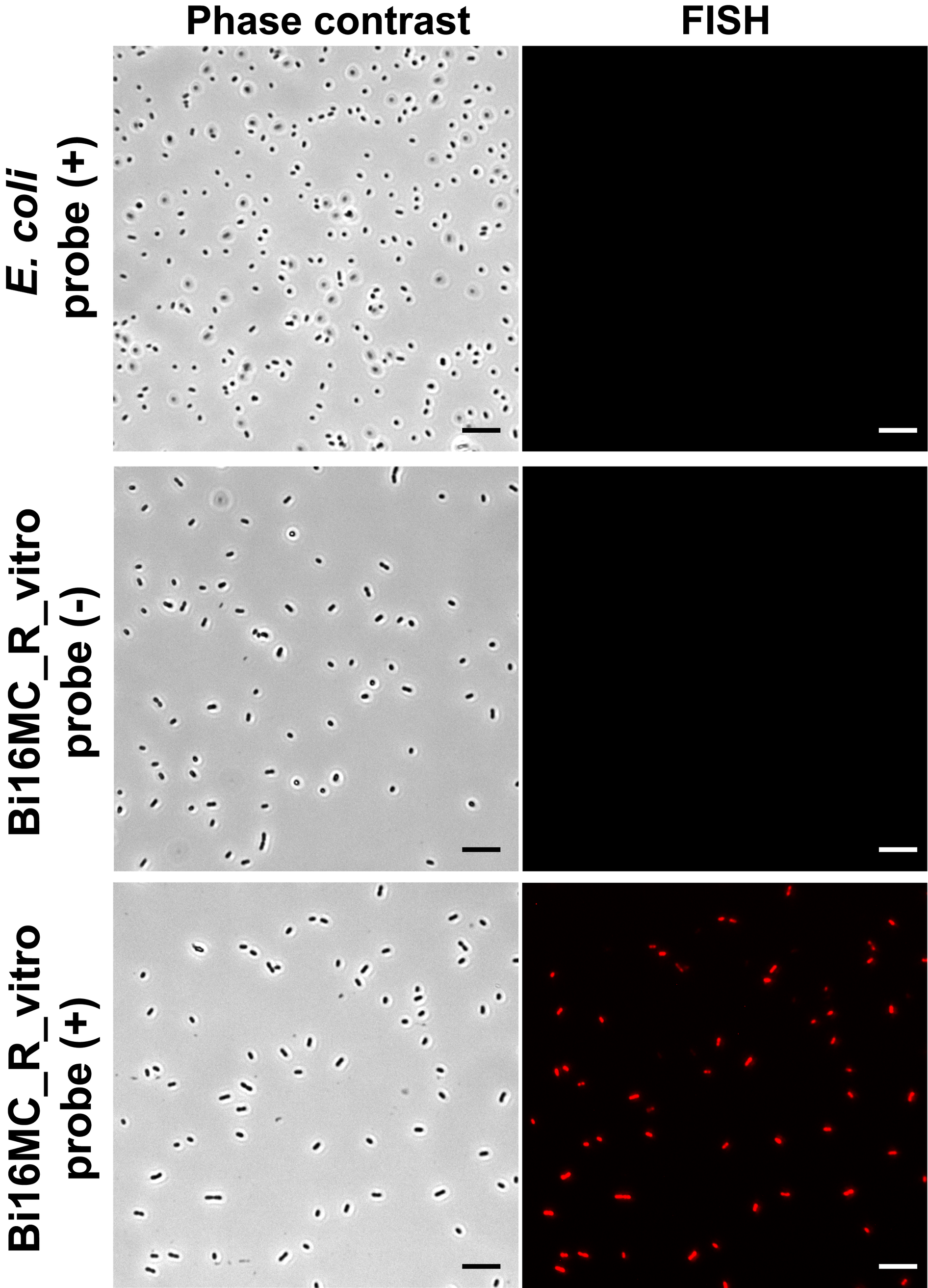

Supplement: S2 Fig — Phase contrast and epifluorescence (FISH of the same areas) micrographs confirm the specificity of FISH probe (Alsym16S) against the culturable Burkholderia Bi16MC_R_vitro isolate from crypts of Blissus insularis, but not against E. coli or the non-probe control. Scale bars = 10 μm. (TIF) [file pone.0161699.s002.tif]

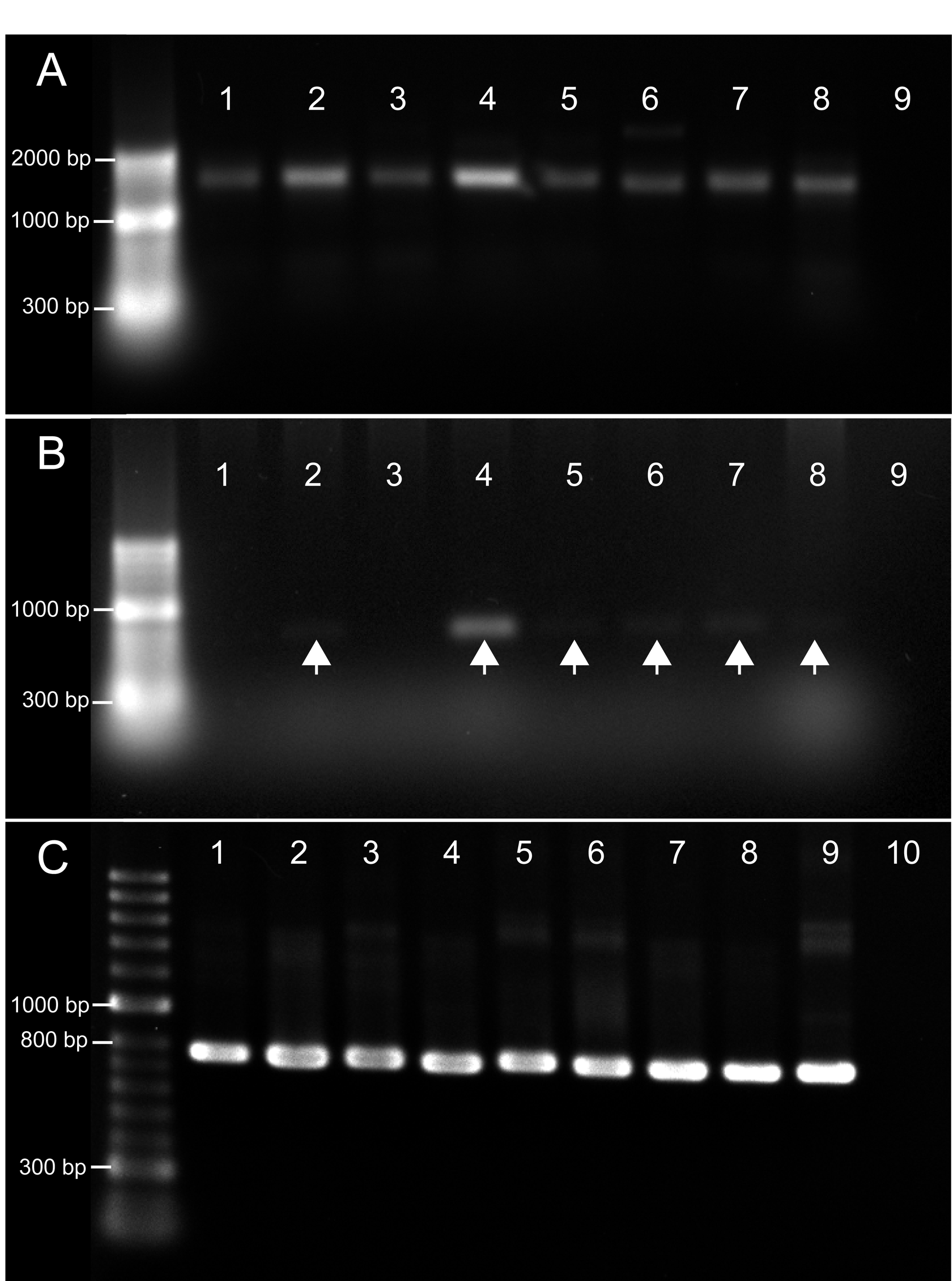

Supplement: S3 Fig — Initial PCR amplifications of the universal 16S rRNA gene (~1.5 kb) (A) and the Burkholderia 16S rRNA gene (~750 bp) (B) detected in the genomic DNA of reproductive tracts from different females. Lanes in panels A and B: 1–5 = Bi09RT_R to Bi13RT_R; 6–8 = Bi09RT_S to Bi11RT_S; 9 = non-template control. The arrows in panel B indicate the faint bands of target Burkholderia 16S rRNA amplicons. (C) The subsequent reamplifications of Burkholderia 16S rRNA gene in the purified universal 16S rRNA amplicons detected in all examined reproductive tract samples. Lanes in panel C: 1–5 = Bi09RT_R to Bi13RT_R; 6–8 = Bi09RT_S to Bi11RT_S; 9 = Bi10MC_R served as a positive control; 10 = non-template control. Standard markers are HyperLadder™ II (Bioline, Taunton, MA). (TIF) [file pone.0161699.s003.tif]

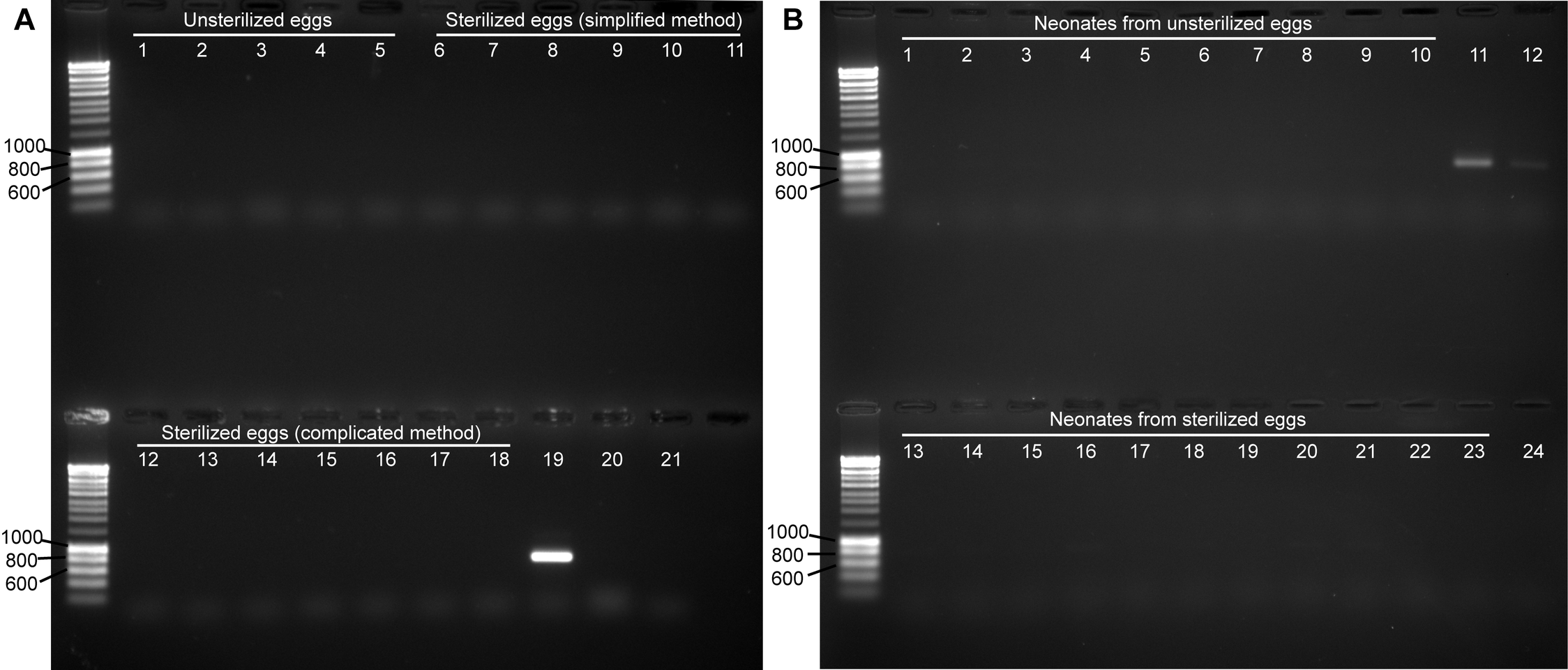

Supplement: S5 Fig — (A) The initial PCR amplifications of the Burkholderia 16S rRNA gene (~750 bp) did not detect Burkholderia in the genomic DNA of eggs, which were from two pairs of B. insularis adults, regardless of the surface sterilization treatment. Lanes 1–5 = five eggs that were not surface-sterilized; 6–11 = six eggs that were sterilized using a simplified method (see detail in Materials and Methods section); 12–18 = seven eggs that were sterilized using a complicated method; 19 = crypt genomic DNA of a female parent; 20 = reproductive tract genomic DNA of a female parent; 21 = non-template control. (B) The initial PCR amplifications of the Burkholderia 16S rRNA gene did not detect Burkholderia in the genomic DNA of neonates (less than 24-hour old), which were from five pairs of B. insularis adults, regardless of the egg surface sterilization treatment. Lanes 1–10 = ten neonates that were hatched from not surface-sterilized eggs; 11 = crypt genomic DNA of a female parent; 12 = reproductive tract genomic DNA of a female parent; 13–23 = eleven neonates that were hatched from sterilized eggs; 24 = non-template control. Standard markers are HyperLadder™ I (Bioline, Taunton, MA). (TIF) [file pone.0161699.s005.tif]

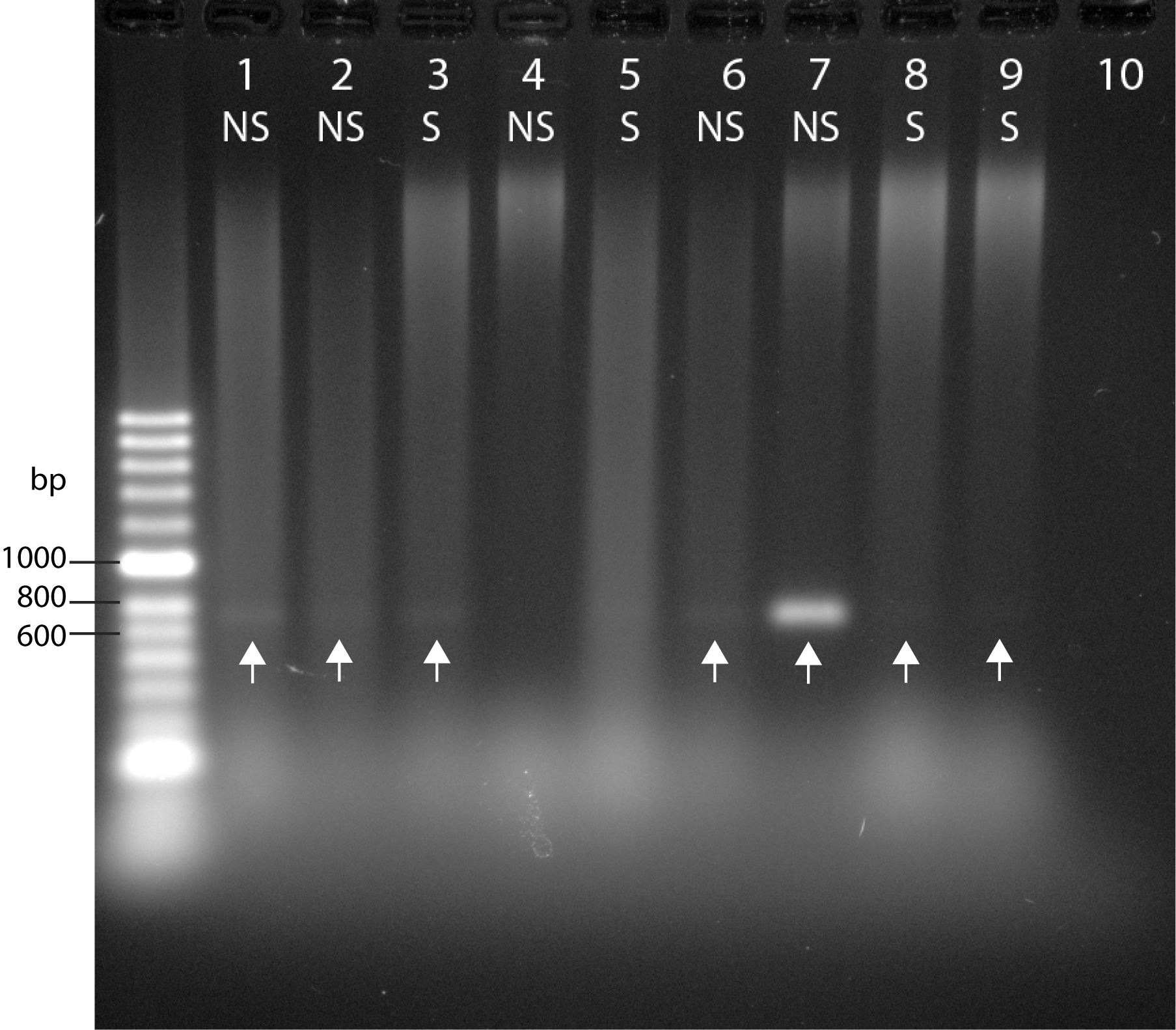

Supplement: S6 Fig — The initial PCR amplifications of Burkholderia 16S rRNA gene detected target amplicons (~750 bp; indicated by arrows) in the genomic DNA from St. Augustinegrass stems of three cultivars. Lanes: 1–2 = donated ‘Floratam’; 3–4 = donated ‘Palmetto’; 5–6 = donated ‘Captiva’; 7–9 = rearing-used ‘Floratam’; 10 = non-template control. Standard marker is HyperLadder™ I (Bioline, Taunton, MA). NS, non-surface-sterilized; S, surface-sterilized. (TIF) [file pone.0161699.s006.tif]

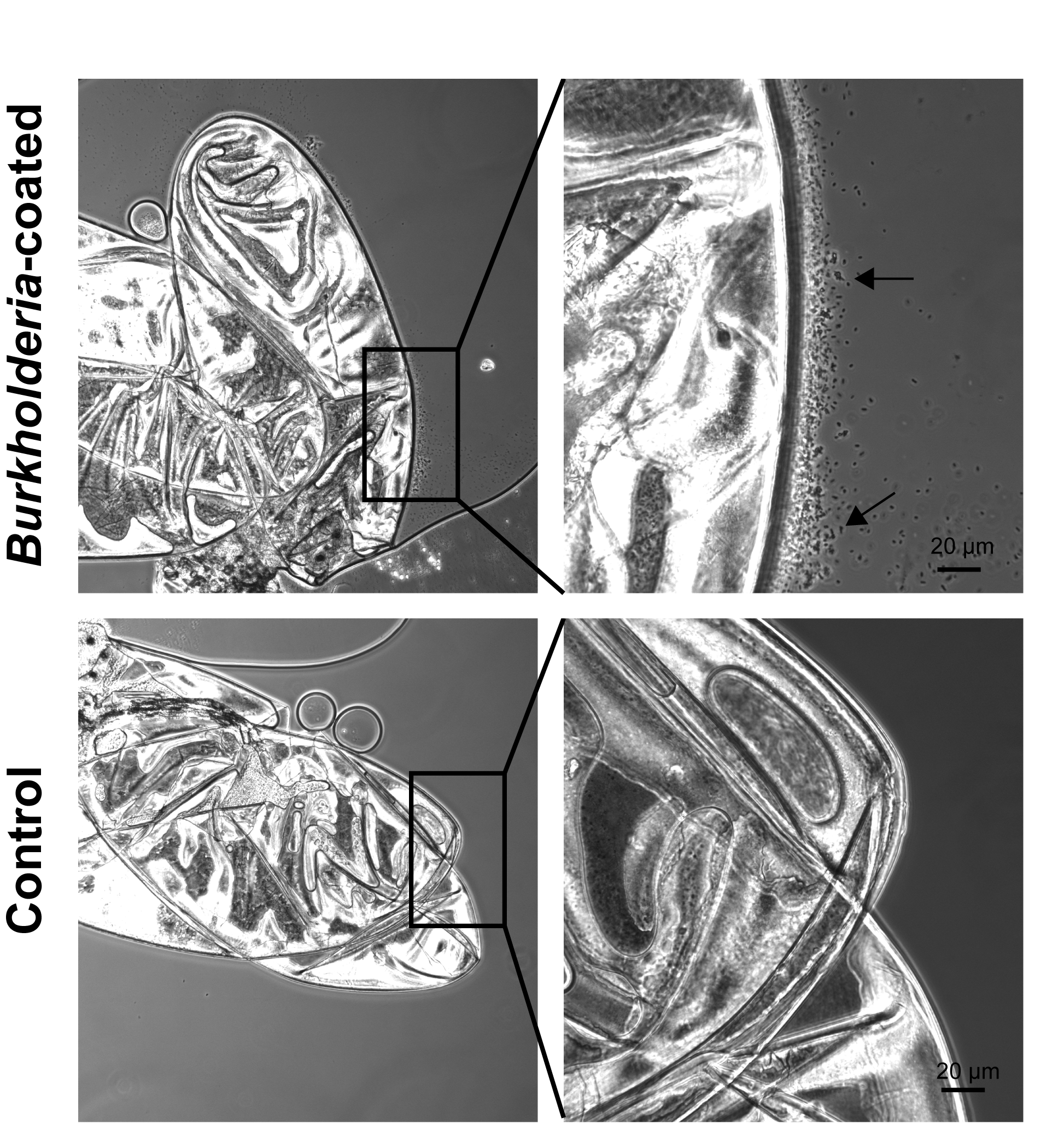

Supplement: S7 Fig — The Burkholderia-coated and uncoated egg chorions were left over by the newly hatched Blissus insularis neonates, which were used in the rearing of neonates on live plants with cultured symbionts experiment. In the Burkholderia-coated treatment, the presence of rod-shaped bacteria on the surface of egg chorion was observed after 5-day of application of Burkholderia suspension (1× 10^9^ cells mL-1). No bacterium was found in the control group. (TIF) [file pone.0161699.s007.tif]

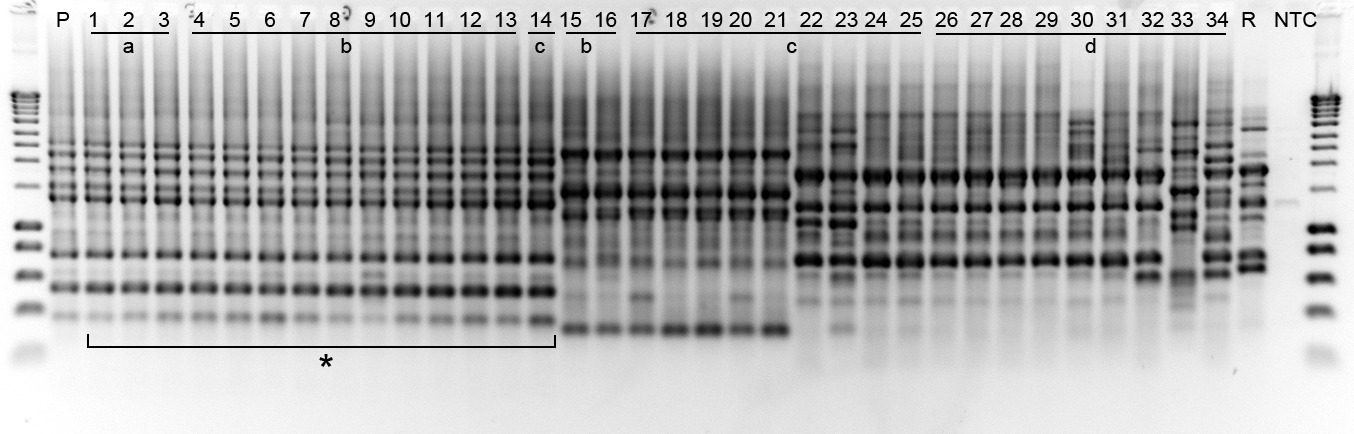

Supplement: S8 Fig — Lanes 1–34 indicate the B. insularis reared on live plants with or without cultured Burkholderia inoculation. Abbreviations: P = the positive control (the inoculated Burkholderia isolate, Bi16MC_R_vitro); a = the treatment chorion+/plant+ (see text for details); b = the treatment chorion-/plant+; c = the treatment chorion+/plant-; d = treatment chorion-/plant-. R = the reference cultured Burkholderia isolate (Bi24MC_R_vitro). NTC = non-template control. Standard markers are HyperLadder™ I. Star indicates the lane-based samples that had ≥75% similarity with the positive control (P). (TIF) [file pone.0161699.s008.tif]
