# Supplementary material for: Environmental Transmission of the Gut Symbiont Burkholderia to Phloem-Feeding Blissus insularis
Source: PLoS One. 2016 Aug 22;11(8):e0161699. doi: 10.1371/journal.pone.0161699 (PMC4993365; doi:10.1371/journal.pone.0161699)
Supplement: S4 Fig — (DOCX) [file pone.0161699.s004.docx]

**S4 Fig. Phylogenetic relationship of *Burkholderia* obtained from *Blissus insularis* females.**

The tree was generated from midgut crypts (denoted as Bi01MC to Bi13MC) and reproductive tracts (denoted as Bi01RT to Bi13RT) on the basis of 705-bp 16S rRNA gene sequences. The sequences denoted as R and S are obtained from two separate *B. insularis* colonies [1]. Sequences detected in the current study are shown in bold. Numbers at the tree nodes represent the maximum-likelihood bootstrap values obtained after 100 repetitions; only values over 50 are shown. In brackets are shown nucleotide sequence accession numbers in the GenBank. Clear and gray circles denote the *Burkholderia* isolates detected in the *B. insularis* field populations [2] and in *C. saccharivorus* [3], respectively; squares denote the *Burkholderia* isolates detected in other heteropteran hosts; stars denote the pesticide-degrading strains. The clades SBE, PBE, and BCC correspond to those described in references [3], [4], and [5], respectively. The clades PC and SC represent pathogen clade and soil clade, respectively.

**Supplementary References**

1. Xu Y, Buss EA, Boucias DG. Culturing and characterization of the gut symbiont *Burkholderia* from the Southern chinch bug, *Blissus insularis* (Hemiptera: Blissidae). Appl Environ Microbiol. 2016;82: 3319–3330. doi:10.1128/AEM.00367-16

2. Boucias DG, Garcia-Maruniak A, Cherry R, Lu H, Maruniak JE, Lietze VU. Detection and characterization of bacterial symbionts in the heteropteran, *Blissus insularis*. FEMS Microbiol Ecol. 2012;82: 629–641. doi:10.1111/j.1574-6941.2012.01433.x

3. Itoh H, Aita M, Nagayama A, Meng XY, Kamagata Y, Navarro R, et al. Evidence of environmental and vertical transmission of *Burkholderia* symbionts in the oriental chinch bug, *Cavelerius saccharivorus* (Heteroptera: Blissidae). Appl Environ Microbiol. 2014;80: 5974–5983. doi:10.1128/AEM.01087-14

4. Suárez-Moreno ZR, Caballero-Mellado J, Coutinho BG, Mendonça-Previato L, James EK, Venturi V. Common features of environmental and potentially beneficial plant-associated *Burkholderia*. Microb Ecol. 2012;63: 249–266. doi:10.1007/s00248-011-9929-1

5. Coenye T, Vandamme P, Govan JRW, Lipuma JJ. Taxonomy and identification of the *Burkholderia cepacia* complex. J Clin Microbiol. 2001;39: 3427–3436. doi:10.1128/JCM.39.10.3427
