# Supplementary material for: Environmental Transmission of the Gut Symbiont Burkholderia to Phloem-Feeding Blissus insularis
Source: PLoS One. 2016 Aug 22;11(8):e0161699. doi: 10.1371/journal.pone.0161699 (PMC4993365; doi:10.1371/journal.pone.0161699)
Supplement: S1 Table — (DOCX) [file pone.0161699.s009.docx]

**S1 Table. The single-nucleotide polymorphisms detected in *Blissus insularis*.**

| Colony | Identity | SNPs *^a^* (bp) | Percentage of SNPs *^b^* | Hypervariable regions *^c^* |
| --- | --- | --- | --- | --- |
| BiR | Bi01MC_R  Bi01RT_R | 0 | 0 | N/A*^d^* |
|  | Bi02MC_R  Bi02RT_R | 1 | 0.1 | V7 |
|  | Bi03MC_R  Bi03RT_R | 6 | 0.8 | V6 |
|  | Bi04MC_R  Bi04RT_R | 0 | 0 | N/A |
|  | Bi05MC_R  Bi05RT_R | 0 | 0 | N/A |
|  | Bi06MC_R  Bi06RT_R | 7 | 1.0 | V3-4, V6 |
|  | Bi07MC_R  Bi07RT_R | 26 | 3.5 | V3-4, V6-7 |
|  | Bi08MC_R  Bi08RT_R | 29 | 4.0 | V3-4, V6 |
|  | Bi09MC_R  Bi09RT_R | 26 | 3.5 | V3-4, V6 |
|  | Bi10MC_R  Bi10RT_R | 9 | 1.2 | V3-4, V6 |
|  | Bi11MC_R  Bi11RT_R | 0 | 0 | N/A |
|  | Bi12MC_R  Bi12RT_R | 0 | 0 | N/A |
|  | Bi13MC_R  Bi13RT_R | 38 | 5.2 | V3-6 |
| BiS | Bi01MC_S  Bi01RT_S | 1 | 0.1 | V7 |
|  | Bi03MC_S  Bi03RT_S | 23 | 3.1 | V3-4, V6 |
|  | Bi04MC_S  Bi04RT_S | 32 | 4.4 | V3-4, V6 |
|  | Bi05MC_S  Bi05RT_S | 20 | 2.7 | V3-4, V6 |

**S1 Table.** Continued.

| Colony | Identity | SNPs *^a^* (bp) | Percentage of SNPs *^b^* | Hypervariable regions *^c^* |
| --- | --- | --- | --- | --- |
| BiS | Bi06MC_S  Bi06RT_S | 6 | 0.8 | V3, V6 |
|  | Bi07MC_S  Bi07RT_S | 3 | 0.4 | V3, V6 |
|  | Bi08MC_S  Bi08RT_S | 29 | 3.9 | V3-4, V6 |
|  | Bi10MC_S  Bi10RT_S | 0 | 0 | N/A |
|  | Bi11MC_S  Bi11RT_S | 0 | 0 | N/A |

*^a^* The single-nucleotide polymorphisms (SNPs) detected using pairwise alignments of the *Burkholderia* 16S rRNA gene sequences generated from midgut crypt- (MC) and reproductive tract- (RT) associated bacteria isolated from each individual female *B. insularis.*

*^b^* Percentage of the SNPs in the respective ~700-bp *Burkholderia* 16S rRNA gene sequences.

*^c^* The hypervariable regions [1] where the SNPs are detected in the 16S rRNA gene sequence.

*^d^* N/A = not available.

**Supplementary References**

1. Xu Y, Buss EA, Boucias DG. Culturing and characterization of the gut symbiont *Burkholderia* from the Southern chinch bug, *Blissus insularis* (Hemiptera: Blissidae). Appl Environ Microbiol. 2016;82: 3319–3330. doi:10.1128/AEM.00367-16
