# Supplementary material for: Environmental Transmission of the Gut Symbiont Burkholderia to Phloem-Feeding Blissus insularis
Source: PLoS One. 2016 Aug 22;11(8):e0161699. doi: 10.1371/journal.pone.0161699 (PMC4993365; doi:10.1371/journal.pone.0161699)
Supplement: S2 Table — (DOCX) [file pone.0161699.s010.docx]

**S2 Table. Putative phylogenetic affiliation of the 16S rRNA gene clones obtained from the reproductive tracts of four BiR and four BiS *Blissus insularis* females.**

| Colony | Closely related taxa identified using the RDP database  (accession no. in GenBank) | | No. of clones | Sequence similarity (%) with | | |
| --- | --- | --- | --- | --- | --- | --- |
|  |  |  |  | closely related taxa *^a^* | pooled RT samples *^b^* | MC samples *^c^* |
| BiR | *β*-proteobacteria | *Burkholderia* sp. (FJ025136) | 7 | 99-100 | 99-100 with Bi02RT_R | 99-100 with Bi02MC_R |
|  |  | *Burkholderia* sp. (AF408997) | 2 | 99 | 97-100 with Bi10RT_R | 97-99 with Bi10MC_R |
|  |  | *Burkholderia* sp. (AF311971) | 1 | 100 | 97-100 with Bi10RT_R | 97-99 with Bi10MC_R |
|  |  | *Burkholderia* sp. (JQ518342) | 1 | 99 | 97-100 with Bi10RT_R | 97-99 with Bi10MC_R |
|  |  | *Burkholderia* sp. (AB665360) | 1 | 99 | 99 with Bi07RT_R | 96 with Bi07MC_R |
|  | *γ*-proteobacteria | *Escherichia* spp. (U00006, CP000948) | 28 | 99-100 |  |  |
|  |  | *Morganella* sp. (AJ301681) | 1 | 99 |  |  |
|  |  | Xanthomonadaceae (EF608545) | 3 | 100 |  |  |
|  |  | Unclassified | 2 | 92-99 |  |  |
|  | *α-*proteobacteria | *Sphingomonas* sp. (AB018439) | 1 | 98 |  |  |
| BiS | *β*-proteobacteria | *Burkholderia* sp. (DQ355168) | 7 | 99-100 | 99-100 with Bi10RT_S | 99-100 with Bi10MC_S |

**S2 Table.** Continued.

| Colony | Closely related taxa identified using the RDP database  (accession no. in GenBank) | | No. of clones | Sequence similarity (%) with | | |
| --- | --- | --- | --- | --- | --- | --- |
|  |  |  |  | closely related taxa *^a^* | pooled RT samples *^b^* | MC samples *^c^* |
| BiS | *β*-proteobacteria | *Burkholderia* sp. (JQ518347) | 2 | 99 | 99 with Bi11RT_S | 99 with Bi11MC_S |
|  |  | *Burkholderia* sp. (AB212227) | 1 | 100 | 99 with Bi11RT_S | 99 with Bi11MC_S |
|  |  | *Burkholderia* sp. (GU144372) | 1 | 100 | 100 with Bi01RT_S | 100 with Bi01MC_S |
|  | *γ*-proteobacteria | *Escherichia* spp. (U00006, CP000948) | 31 | 99-100 |  |  |
|  |  | *Morganella* sp. (AB089244) | 1 | 99 |  |  |
|  |  | Xanthomonadaceae (EF608545) | 1 | 99 |  |  |
|  |  | *Serratia* sp. (AY700617) | 1 | 99 |  |  |
|  |  | Pseudomonadaceae (EF608541) | 1 | 99 |  |  |
|  | Bacteroidetes | *Myroides* sp. (M58777) | 1 | 99 |  |  |

*^a^* The sequence similarity was determined by comparing the ~900-bp 16S rRNA gene sequences of clones against the closely related sequences published in the Ribosomal Database Project (RDP).

*^b^* The sequence similarity was determined by the pairwise alignments of 400- to 500-bp 16S rRNA gene sequences between the clones and pooled *Burkholderia* 16S rRNA gene generated from the female reproductive tracts.

*^c^* The sequence similarity was determined by the pairwise alignments of 700- to 900-bp 16S rRNA gene sequences between the clones and universal 16S rRNA gene generated from the corresponding female midgut crypt.
